# Immune biological rationales for the design of combined radio- and immunotherapies

**DOI:** 10.1007/s00262-019-02460-3

**Published:** 2020-01-18

**Authors:** Michael Hader, Benjamin Frey, Rainer Fietkau, Markus Hecht, Udo S. Gaipl

**Affiliations:** Department of Radiation Oncology, Universitätsklinikum Erlangen, Friedrich-Alexander-Universität Erlangen-Nürnberg (FAU), Universitätsstraße 27, 91054 Erlangen, Germany

**Keywords:** Radioimmunotherapy, Hyperthermia, Immune checkpoint inhibitors, Tumor microenvironment, Personalized medicine, CITIM 2019

## Abstract

Cancer immunotherapies are promising treatments for many forms of cancer. Nevertheless, the response rates to, e.g., immune checkpoint inhibitors (ICI), are still in low double-digit percentage. This calls for further therapy optimization that should take into account combination of immunotherapies with classical tumor therapies such as radiotherapy. By designing multimodal approaches, immune modulatory properties of certain radiation schemes, additional immune modulation by immunotherapy with ICI and hyperthermia, as well as patient stratification based on genetic and immune constitutions have to be considered. In this context, both the tumor and its microenvironment including cells of the innate and adaptive immune system have to be viewed in synopsis. Knowledge of immune activation and immune suppression by radiation is the basis for well-elaborated addition of certain immunotherapies. In this review, the focus is set on additional immune stimulation by hyperthermia and restoration of an immune response by ICI. The impact of radiation dose and fractionation on immune modulation in multimodal settings has to be considered, as the dynamics of the immune response and the timing between radiotherapy and immunotherapy. Another big challenge is the patient stratification that should be based on matrices of biomarkers, taking into account genetics, proteomics, radiomics, and “immunomics”. One key aim is to turn immunological “cold” tumors into “hot” tumors, and to eliminate barriers of immune-suppressed or immune-excluded tumors. Comprehensive knowledge of immune alterations induced by radiation and immunotherapy when being applied together should be utilized for patient-adapted treatment planning and testing of innovative tumor therapies within clinical trials.

## Introduction

Starting from antiquity until the 1980s, cancer was defined as a cellular disease caused by an invasion of abnormal cells into healthy tissue. Therefore, standard of care was to remove all cancer cells by surgical techniques, radiotherapy, and/or cytotoxic agents, referred to as “chemotherapeutics” [1]. As a result of ongoing research, findings about genetic mutations and epigenetic deviations from healthy cells improved the treatment concept. Cancer should be treated with customized drugs in an individualized manner [2]. Nevertheless, cancer was still seen as a local disease with the three consecutive steps of formation, progress, and finally uncontrolled growth [3]. Following, in 2002, the cancer immune editing concept has been worked out. This so-called 3E hypothesis is connecting initial *e*limination of malignant cells by innate and adaptive immune cells, *e*quilibrium status in which tumor cells acquire further mutations in an immune-mediated tumor dormancy, and finally an *e*scape of cancer cells from immune surveillance [4]. The *e*scape phase is defined by essential characteristics, which belong also to the so-called hallmarks of cancer, i.e., cancer cells appear non-immunogenic during immunoediting phase or actively suppress the immune system [5]. Therefore, a regain of anti-cancer-immunosurveillance using additional immunotherapies such as anti-programmed death ligand 1 (PD-L1)/PD-1 and/or anti-cytotoxic T-lymphocyte associated protein 4 (CTLA-4) [6] and immune stimulants such as hyperthermia [7, 8] in multimodal treatment settings is a further step forward to get the cancer under control and to cure it. However, immune biological rationales are very important for the design of multimodal therapies including radiotherapy and immunotherapy, which is content of this review.

## Tumor microenvironment and its immune cells

The tumor microenvironment (TME) is a spatially organized, very complex and dynamic ecosystem with different cellular components. The latter include mainly the tumor, supporting cells, e.g., fibroblasts and endothelial cells, as well as cells of the immune system [9, 14]. Within the immune cell subpopulations, there can be found cells that are associated with acute inflammation, e.g., neutrophils, basophils, and eosinophils, cells of the innate immune response [e.g., natural killer (NK) cells and dendritic cells (DCs)], and cells from the adaptive immune response (e.g., T cells and B cells). Spatially regarded, T lymphocytes and macrophages are located both in the center and at the invasive margins of the tumor, whereas DCs as well as B lymphocytes primarily infiltrate tumor-adjacent lymphoid islets (TLSs) [10, 11, 14].

### Sophisticated techniques in tumor microenvironment analysis

Within the last 2 decades, knowledge about the correlation between tumor microenvironment composition and the tumor tissue has increased significantly by a number of analytical techniques such as immunofluorescence (IF), immunohistochemistry (IHC), immunocytochemistry (ICC), as well as immunophenotyping (IPT). A new innovative technique is the so-called microenvironment cell populations (MCP) counter which gives very precise quantitative information about the cell content of immune and stromal cell populations in heterogeneous tissue samples from transcriptome data. Using MCP counter in 25 different cancers (*n* = 19,000), infiltration of CD8^+^ T cells could be correlated with favorable prognosis and the relative multifaceted cellular composition of the tumor microenvironment was shown in different cancers [12–15].

### Function of different immune cells

- *DC* In the 1970s, the Nobel Laureate Ralph Steinman and his colleague Zanvil Cohn discovered a cell type that they coined DCs. Under physiological conditions, the main function of DCs is to build a conjunction between the innate and adaptive immune response as they engulf antigens. As soon as they are exposed to danger signals or other activation signals, they mature and become activated and prime naïve T or B cells inside lymph nodes. DCs, as they have many phenotypes for an effective activation of the adaptive immune system, express a series of activatory and inhibitory receptors [16]. Furthermore, DCs can produce numerous pro-inflammatory or immunosuppressive cytokines. Interestingly, tumor cells can inhibit DC maturation and functionality. Nevertheless, high level of mDCs in the tumor and its microenvironment are associated with good clinical outcome in certain cancers [17].
- *T lymphocytes* These cells of the adaptive immune system are responsible for the destruction of mutated cells as well as intracellular invaders, e.g., bacteria and viruses. Thus, T lymphocytes are essential for the cell-mediated immune response of adaptive immunity. According to their main surface (co-)receptors, a first T-cell subgroup classification into CD3^+^/CD4^+^ (T helper cells) and CD3^+^/CD8^+^ (cytotoxic T lymphocytes) is appropriate. T helper cells recognize a region of MHC class II protein, and cytotoxic T lymphocytes MHC class I proteins. Due to the fact that T lymphocytes are essential in adaptive immunity and tumor elimination, they can be considered as prognostic markers [9, 18]. For example in melanoma, head and neck, breast, bladder, urothelial, ovarian, colorectal, and lung cancer, a high density of CD3^+^ T cells, CD8^+^ cytotoxic T cells, and memory T cells (CD45RO^+^) was correlated with favorable disease-free survival (DFS) and overall survival (OS) [4, 12, 19], as well as a lower probability of metastatic spread and progression-free survival (PFS) [10, 20, 21] with a few exceptions, e.g., in clear cell renal cell carcinoma (ccRCC) [17, 22–24]. A first subgroup analysis of this ccRCC entity was reported by Giraldo et al. While patients with “normal” oligoclonal CD8^+^ T-cell texture had a good clinical outcome, patients with polyclonal CD8^+^ T-cell texture showed a limited cytotoxic capacity and presumably did not recognize any relevant tumor-associated antigens (TAAs) [24]. These results emphasize that both the tumor type and the TME including its immune cell (sub)populations have an impact on prognosis and clinical outcome.
- *NK cells* They are large lymphocyte-like cells of the innate immune system whose primary function is the early defense against both allogenic (nonself) cells and autologous transformed cells, e.g., tumor cells and cells infected with viruses, bacteria, or parasites. This makes NK cells a good prognostic factor for clinical outcome, especially in the context of recurrences [12, 25, 26].
- *B cells* These cells of the adaptive immune system are of central importance in human immunity as they produce immunoglobulins (antibodies). In a first step, antigens are encountered by a B cell receptor. This turns naïve mature B cells into activated B cells that can proliferate, differentiate into plasma cells, and finally produce antibodies. As for T lymphocytes, a summary by Vano et al. [14] shows that a high density of B cells within the TME can be correlated with a better prognosis including breast cancer [27], NSCLC [28] or head and neck cancer [29], whereas the database of B cells in the context of cancer is still scarce. However, some mechanistically explanations underline the positive role of B cells in the anti-tumor immune response as B cells can activate DCs or present antigens for an initial priming and expansion of CD4^+^ [30] and CD8^+^ T cells [31]. However, in this context, B cells may also play a negative role in anti-tumor immune response as they maintain a chronic inflammation [32] by the promotion of neo-angiogenesis [33], and/or by direct blocking of cytotoxic T-cell responses [34].
- *Macrophages* These phagocytes exist in almost all tissues and develop both during embryonic phase as well as during the life span in the bone marrow. The mature forms of macrophages migrate into tissues, where a differentiation takes place. Macrophages engulf and destroy invading microorganisms as well as infected cells and pathogens. This makes them an important player in innate immune responses as they produce many cytokines and chemokines. They are central in initiation and termination of inflammation [35]. Macrophages release, e.g., the mostly immunosuppressive transforming growth factor (TGF)-β. The latter suppresses cytotoxic T cells (CD8^+^), pushes the differentiation of naïve CD4^+^ T cells to regulatory T cells (Tregs), and polarizes macrophages to a M2 phenotype. In general, inflammation has many positive effects that are described by Latin words calor, dolor, rubor, and tumor, meaning heat, pain, redness, and swelling. The phases of an inflammation are described by a recruitment of macrophages and neutrophils, followed by monocytes which rapidly differentiate into macrophages. Finally, if the inflammation continues, eosinophils migrate into the tissue. Within the tumor core and the invasive margin, the so-called tumor-associated macrophages (TAMs) are a major component. As already described for B and T cells, TAMs have tumor-specific positive or negative prognostic relevance. Positive prognosis was seen for example in prostate [36] or cervical cancer [37] in contrast to negative prognosis in breast [38], melanoma [39], and ovarian cancer [40]. An explanation for these discrepancies might be a switch in the phenotype from a more anti-tumoral one (M1) to a more pro-tumoral one (M2) and vice versa [48].

## Immune modulatory effects of radiation

### Targeted, non-targeted, and abscopal effects

Ionizing radiation (hereinafter: radiation) has both direct effects on DNA molecules and indirect effects via reactive oxygen or nitrogen species (RONS) that damage cell components like the DNA. Hence, until mid-2000s, radiation was considered to act only “targeted” on the cell, leading to cell-cycle arrest, and finally to cell death [41]. A paradigm shift has then taken place in the field of radiotherapy since “non-targeted” effects were described. These effects include not only the tumor, but also its microenvironment including bystander (5 mm), as well as nearby tissue (5 cm) [42], and the whole organism [43, 44]. “Non-targeted” effects are connected on one hand by communications between irradiated and non-irradiated cells close to each other and on the other hand by a release of signal molecules such as cytokines, chemokines, and damage-associated molecular patterns (DAMPs) into surrounding tissue [42, 45]. Besides “non-targeted” effects of radiation, also “off targeted” effects, which are better known as abscopal effects, do exist. Since abscopal effects occur out of the irradiation field, they are mostly related to systemic immune-mediated effects [46, 47].

### Immune activatory effects by radiation

Historically, radiotherapy was considered to have only immunosuppressive effects. Prominent examples are the downregulation of the immune system for allogenic transplantation and the reduction of co-stimulatory surface markers (CD80 and CD86) on immature DCs, thus inhibiting T-cell activation [48]. Nowadays, it is well accepted that radiotherapy also induces immune activation. After radiotherapy, the expression of MHC molecules, stress ligands, adhesion molecules, death receptors, and ligands increase on tumor cells [45]. Furthermore, radiotherapy causes different cell-death modalities, such as apoptosis, necro(pto)sis, mitotic catastrophe, or senescence [49]. This leads to a spatiotemporal release of DAMPs that attract and activate cells of the innate and cells of the adaptive immune system. One prominent danger signal is heat shock protein 70 (HSP70). Furthermore, DAMPs are high-mobility group box 1 (HMGB1) protein, adenosine triphosphate (ATP), as well as DNA and RNA [50]. Interferons (IFNs) type I and II are induced by DAMPs. Type I interferons (INF-α and/or IFN-β) stimulate DCs to cross-present antigens, whereby clonal expansion of T cells is enhanced which finally results in tumor-specific T-cell responses [51]. Type II interferon (IFN-γ) is secreted by activated T cells and NK cells, and influences vasculature for immune cell trafficking and immune recognition [52]. Not fully explained remains the question, whether radiotherapy alone has the potential to act as a kind of in situ cancer vaccine [53].

### Immunosuppressive effects by radiation

Each day, around 50–70 billion cells undergo programmed cell death (PCD), better known as apoptosis, which is necessary to self-renew, e.g., tissue or bone marrow. Thus, it is not surprising that this huge number of apoptotic cells is normally cleared by phagocytosis without inflammatory reactions or tissue scarring. Apoptotic tumor cells might, therefore, also foster immune suppression [54]. Tumor cells can further secrete TGF-β or increase the expression of immune suppressive checkpoint molecules such as PD-L1 to make their microenvironment immunosuppressive; radiation even augments these effects [55–57]. Furthermore, radiation can increase the level of chemokines like CCL2 that attracts monocytes into the tumor and its microenvironment. In the latter, differentiation of monocytes into TAMs occurs [58]. TAMs are of great importance in inflammation and immunosuppressive processes which are described above. Another mechanism to suppress immune responses is the upregulation of immune checkpoint molecules (ICM) on tumor cells and on tumor-infiltrating immune cells [59, 60]. The ICM expression is very dynamic and is influenced by tumor treatment modalities [57, 61]. Furthermore, radiation further induces immune suppression by locally and systemically killing of immune cells. Generally, lymphocytes are more radiosensitive than macrophages, DCs, or NK cells [62].

### Fractionation scheme and the impact of radiation dose on immune modulation

If there is any perfect immunogenic radiation dose and fractionation scheme, it has not been found yet. This is amongst reasons like tumor size and/or genetic signature related to the dynamics of immune responses [45, 63]. With a radiation dose of 1.8–2.0 Gray (Gy) per day, classical normofractionation is applied five times a week over 3–7 weeks for treatment of solid tumors. Hypofractionation, in contrast, is any dose exceeding 2.0 Gray per day. This increases quality of life of the patients, since they do not have to come for irradiation every day and might even result in better outcome with equivalent or even less side effects, as already demonstrated for breast cancer [64]. Starting from very low single doses of 0.5–1.0 Gy and a total dose of 3.0–6.0 Gy, immunosuppressive effects of radiation, e.g., on macrophages can be found [65]. However, still low cumulative doses of 10 Gy (5 × 2 Gy) are capable of generating an immunostimulatory macrophage phenotype [66]. Single high radiation doses (e.g., 1 × 20 Gy) as they are applied in radiosurgery significantly boosted activation and maturation of DCs [67]. However, under distinct conditions, too high single doses may again dampen the immune response by triggering the expression of repair exonucleases such as TREX-1 that degrade radiation-induced immunogenic DNA [68, 69]. Nevertheless, Filatenkov et al. discovered in a murine model that a high single dose of 1 × 30 Gy induced a significantly higher infiltration of cytotoxic CD8^+^ T cells into tumors in comparison to hypofractionated irradiation with 10 × 3 Gy [70]. Another in vitro study considered the fractionation/dose correlation between supernatant from tumor cells and the secretion of immune activating cytokines and maturation markers of co-incubated DCs. DCs that had been incubated with supernatant from norm- (5 × 2 Gy) or hypofractionation (3 × 5 Gy) irradiated tumor cells expressed significantly higher levels of maturation markers such as CD80, CD83, and CD25, and secreted significantly higher levels of immune activating cytokines such as IL-12p70, IL-8, IL-6, and TNF-α in comparison to a single-dose irradiation of 1 × 15 Gy [71]. Other studies of dose-dependent immune-stimulatory effects have focused on HMGB1 [72] or on a combination of radiotherapy with checkpoint inhibitors such as anti-PD-L1 [73]. The most immunogenic radiation dose is most likely dependent of many factors (tumor, microenvironment, time of irradiation, and combination with immunotherapy) and alternating fractionations with higher and lower dose per fraction might be tested in future clinical trials, as already suggested in [74] based on immune biological considerations. The latter also include addition of hyperthermia to radiotherapy to improve anti-tumor immune responses [75].

## Hyperthermia as immune modulator

**Fig. 1:**
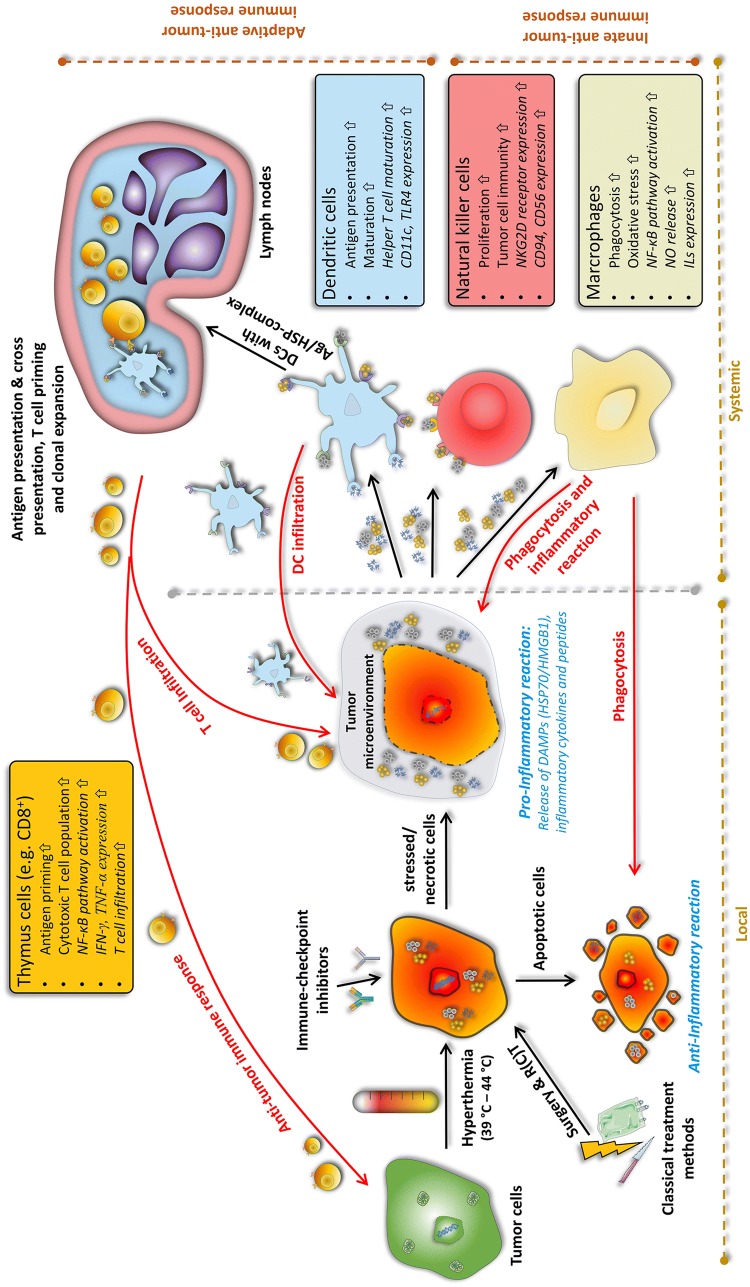
Induction of anti-tumor immune responses by multimodal treatment settings consisting of the classical tumor treatments (surgery and radio(chemo)therapy) combined with immune modulators such as immune checkpoint inhibitors and/or hyperthermia. Depending on the temperature, hyperthermia is capable of inducing both, apoptotic cells and necrotic cells, respectively. Primarily necrotic cells release danger-associated molecular patterns (DAMPs) and inflammatory cytokines into the tumor microenvironment. Dendritic cells (DCs) take up tumor antigens and tumor antigen–DAMP complexes and cross-present it to T cells in lymph nodes. This leads to T-cell priming, clonal expansion, and finally an adaptive anti-tumor immune response against the tumor. Additionally, DAMPs and cytokines can also directly activate natural killer (NK) cells or macrophages as parts of the adaptive immune system. Generally, hyperthermia has several stimulating mechanisms on immune cells, as shown in the colored boxes

Findings of heat effects on our body date back to 5000 BC [76]. Almost 7000 years later, in the 18th and 19th century, correlations between shrinkage of tumors and febrile diseases like malaria were found. Consequently, William B. Coley initiated several studies on inducing heat by *Streptococcus pyogenes* extracts, later called “Coley’s toxin”, in tumor patients [77]. Since the 1980s, the positive effects of heat are utilized in the clinics under the name of hyperthermia. Via an exogenous energy source such as water-filtered IR-A, ultrasound, capacitive or radiative heating, the tumor area is heated up into supraphysiological range between 39 and 45 °C for typically 60 min per session [78]. Hyperthermia is, therefore, not to confuse with thermal ablation at temperatures above 60 °C, which leads to coagulative necrosis [79]. This led to the situation, in contrast to the accepted therapeutic benefit of radiation and/or chemotherapy, that hyperthermia still has a negative reputation caused by insufficient quality control and often non-standardized treatment methods [80]. In this context, it should be stressed that not each hyperthermia heating approach (local/regional/whole-body hyperthermia) is suitable for every tumor (superficial/deep-regional) and ensures the desired temperature profile within the tumor [81, 82]. However, since technical improvements like the development of radiative multi-antenna applicators with sensors for E-field monitoring, real-time temperature monitoring (particularly, online magnetic resonance tomography), and computer-based (online) treatment planning, hyperthermia is more and more accepted as an additive treatment modality in the clinics [78]. These technical improvements are converging with several phases I–III trials which have shown a significant beneficial effect of additive hyperthermia in terms of local control (e.g., malignant melanoma) and survival (e.g., soft-tissue sarcoma) [78, 83]. Besides improving hyperthermia devices and quality monitoring, the field of research is on the optimal (thermal) dose to achieve the maximum benefit with minimal side effects. This includes not only killing the tumor cells but also sensitizing the immune system (Fig. 1) [84]. Combination of radiotherapy with hyperthermia and immune checkpoint inhibitors should, therefore, be tested in the future. Furthermore, thermosensitive liposomes as targeted drug-delivery systems might add well to efficient stimulation of the immune system [85].

### Thermobiological rationale for hyperthermia

The “chief actors” in nearly every biological process are proteins. Heat can change their structural and mechanical function. Specifically, the secondary and tertiary protein structures (α-helix and β-sheet) change and become denaturized and aggregate non-specifically. This causes several intramolecular mismatches, finally resulting in, e.g., a decrease in the number of mitochondria and lysosomes, nucleoli swell, and a deposition of protein complexes. This results in cell death such as mitotic catastrophe [86]. Heat shock affects cell doubling as it also inhibits DNA replication, DNA transcription, mRNA processing, as well as the repair of damaged DNA. To sum up, heat can stop cell growth, silences (senescence) and kills cells [87]. But to be protected from these cell damaging effects, cells have intrinsic mechanism in forms of stress proteins, also called heat shock proteins (HSP). An upregulation of HSP is related to thermo-tolerance. HSP functions as a chaperone for the denaturation of heat-sensitive proteins [88].

It is further accepted that heat inhibits DNA repair by, e.g., inducing the degradation of DNA repair proteins [89, 90]. Since γH2AX, a predictive marker for DNA damage response, respectively, double-strand breaks (DSB), can be imaged by fluorescence staining, quantitative correlations of DNA damage and temperature and length of heat exposure were found [91, 92]. Heat induces DSBs mainly in S-phase of the cell cycle, while radiotherapy does it in G2/M-phase [75, 93].

One has always to be aware of that hyperthermia is applied in a temperature between 39 and 45 °C and different physiological effects happen in this temperature range. The most prominent one that is induced by heat, both in normal and cancerous tissue, is an increased blood flow by expanding the vessels to keep the temperature in a physiological range. Especially, within the chaotic and inefficient vascularized network of tumors, this leads to a re-oxygenation and enhanced infiltration of immune cells [94]. However, it was long controversially discussed, whether hyperthermia activates or suppresses the immune system by inducing tolerances [84]. Nevertheless, it has become clear that synergistic anti-tumor effects can be achieved by combining hyperthermia with other treatment methods, i.e., radio(chemo)therapy and/or ICI (Fig. 1).

We analyzed the additional effect of hyperthermia on tumor cell death forms in different in vitro models. Necrosis was found to be the prominent form of cell death, both under hyperthermia with 41.5 °C alone and significantly more in combination with radiotherapy [95, 96]. As already discussed, necrotic cells are generally pro-inflammatory and immune stimulatory as they lose their membrane integrity and release DAMPs. Higher concentrations of HSP70 and HMGB1, the most prominent danger signals, were found only after combination treatment of radiotherapy and hyperthermia [7, 97]. To mimic the clinical situation, B16-F10 tumor-bearing C57/BL6 mice were treated with combination of hyperthermia and radiotherapy or with the single modalities only. Adding hyperthermia to radiotherapy particularly fostered the infiltration of DCs into the solid tumors [97]. In addition, hyperthermia was demonstrated to increase NK cell-activating surface receptors such as NKG2D and MHC class I-related chain A, making NK cells more active against cancer cells [98]. Furthermore, NK cells seem to be important in the effector phase of tumor killing after treatment with radiotherapy plus hyperthermia [99]. Besides positive effects of adding hyperthermia to radiotherapy, certain chemotherapeutics also benefit from additional heating. Additive effects exist for doxorubicin, ifosfamide, and gemcitabine, while synergistic effects can be found, e.g., for mitomycin C, bleomycin, cisplatin, and carboplatin. Reasons for that are a better perfusion of the tumor tissue, a higher metabolic rate, and better membrane permeability of the cells [100]. We continuously have been focusing both on pre-clinical in vitro and in vivo model systems to examine immune modulations induced by combination of hyperthermia and radiotherapy. We additionally have started to perform longitudinal immunophenotyping of cancer patients who are treated within clinical trials, as, e.g., in the HYCAN-Trial (NCT02369939) for anal carcinoma. First data give hints that particularly cells of the innate immune system do recover faster in the peripheral blood when hyperthermia is added to radiochemotherapy.

## Challenges of cancer immunotherapy

The already mentioned “Coley’s toxin” can be considered as one of the first cancer immunotherapies, as it activated an anti-tumor immune response with pro-inflammatory stimuli [77]. The current approaches of numerous monoclonal antibodies (mAbs) targeting the immune checkpoints PD-1/PD-L1 and CTLA-4 are different as they reactivate a pre-existing anti-tumor immunity. Therefore, immunological “hot” tumor entities with, e.g., a high degree of infiltrating CD8^+^ T cells, and/or high mutational burden like melanoma or non-small cell lung cancer (NSCLC) show good clinical response [101]. Nevertheless, most of the patients still do not respond adequately to immune checkpoint inhibitors. Some explanations are listed in the following.

- *“Cold” and “Hot” tumors* The expression of PD-L1 and the density of CD8^+^ T cells are distinct biomarkers of response to PD-1/PD-L1 antagonists. While “hot” tumors are per definition “T cell infiltrated” and mostly respond to immunological treatment, “cold” tumors are a challenge, as no adequate adaptive immune response occurs [102]. Still under investigation is which step of the anti-cancer immune response is not functional. It could be the absence of T cells (e.g., CD8^+^) within the tumor, a deficit of antigen-presenting cells, i.e., DCs, not enough trafficking of T cells to and into the tumor mass or no adequate T-cell priming/activation [103]. Several reports confirm these thoughts, both in mice experiments [48, 103] and in humans, as, e.g., in melanoma patients [104, 105]. Currently, the CheckRad-CD8 study (NCT03426657) investigates the change of CD8^+^ tumor-infiltrating immune cell density for patient selection after initial chemo-immunotherapy. Well-responding patients with locally advanced HNSCC are consecutively treated with double checkpoint (durvalumab + tremelimumab) blockade and normofractionated radiotherapy. The aim is to replace toxic radiochemotherapy by combination of radiotherapy with immunotherapy in pre-selected patients.
- *The timing between radiotherapy/chemotherapy and immunotherapy* In a pre-clinical study by Dovedi et al., tumor-bearing mice were locally irradiated with 10 Gy in five fractions simulating normofractionated radiotherapy. Anti-PD-L1 was given on the first or last day of irradiation, or 7 days after the last irradiation. While overall survival was not significantly prolonged by sequential treatment, simultaneous checkpoint-blockade revealed a significantly benefit in overall survival [60]. A comparable correlation between therapeutic outcome and the timing between radiotherapy and checkpoint-inhibitor donation (< or > 14 days) was observed in the PACIFIC-Trial by Antonia et al. in NSCLC. Patients who received the checkpoint-inhibitor close by to radiotherapy (< 14 days) profited the most [106]. Currently, a phase II study on small cell lung carcinoma (NCT02046733) is investigating whether additional immune checkpoint inhibitors (Nivolumab/Ipilimumab) improve clinical outcome, even if they are applied 6–8 weeks after the last of four sessions of chemotherapy. However, the perfect scheduling of radiotherapy/chemotherapy and immunotherapy is still a point of discussion, especially in terms of safety and efficacy [107].
- *The dose of radiation* In a pre-clinical study by Grapin et al., CT26 cells in tumor-bearing mice were irradiated with same biologically effective dose of 18 × 2 Gy, 3 × 8 Gy, and 1 × 16.4 Gy, respectively. Additionally, anti-PD-L1 and anti-TIGIT (anti-T-cell immunoreceptor with Ig and ITIM domains) injection over 3 weeks was performed 3 times/week, starting on the first day of irradiation. First, without any antibody, tumor growth delay was higher at 18 × 2 Gy and 3 × 8 Gy in comparison to 1 × 16.4 Gy. Adding only anti-PD1, irradiation with 3 × 8 Gy was the most effective treatment (8/12 remissions). Using both anti-PD-L1 and anti-TIGIT resulted in 9/10 complete remissions with 3 × 8 Gy and 7/12 complete remissions with 18 × 2 Gy [108]. One has to stress that lymph nodes should be spared of irradiation, since tumor-specific CTL are primed there [109, 110].
- *The status of PD*-*L1* Still under investigation and controversially discussed is the correlation between PD-L1 status and clinical outcome of treatment with immune checkpoint inhibitors [111]. Normally, PD-L1 status is diagnosed by antibody staining of biopsies before radio(chemo)therapy and not during or afterwards. Many studies revealed significant benefit of combining radio(chemo)therapy with immunotherapy instead of monotherapy, but a lack of standardized antibody staining protocols, inhomogeneity in the biopsy material, and individual patient history and treatment processes, e.g., different drugs and irradiation schemes/doses, aggravate a clear statement [112, 113]. Positive treatment results of radiotherapy with inhibition of the PD-1/PD-L1 axis might even be influenced by initially PD-L1-negative patient subgroup [114]. However, again, PD-L1 status was just determined at the beginning and not during therapy. This calls for close meshed immune monitoring of patients who do receive multimodal tumor therapies.
- Finally, to achieve higher response rates, a combination with immune-stimulatory methods such as radio(chemo)therapy and other vaccination techniques is under investigation with the encouraging results [115]. Going back to cancer development, whereas the 3E model consists of *e*limination, *e*quilibrium and *e*scape, immunotherapy could be described by the 3R model: *r*everse by, e.g., CTLA-4, *r*ejuvenate by, e.g., CAR-T cells and *r*estore by anti-PD-1/PD-L1 and further tumor-associated immune checkpoint inhibitors [116].

## Multimodal tumor therapy setting: patient-adapted treatment design

Frey et al. demonstrated in a mouse model with syngeneic CT26 tumors that radiation-induced immune cell infiltration into tumors is time- and immune cell-dependent. While macrophages and DCs increasingly infiltrated 5 days after the first irradiation with 2 × 5 Gy, CD8^+^ T cells had a delayed infiltration with a maximum on day 8 until the first irradiation [63]. A comparable time effect on infiltration of CD8^+^ T cells was found by Hettich et al. in a B16 melanoma model with irradiation of the tumor with 24 Gy in 12 consecutive days, i.e., mimicking normofractionated radiotherapy [117]. To protect radiosensitive immune cells and to increase their infiltration, irradiation schemes could be optimized in the future based on such knowledge. It has, however, to be stressed that the function of remaining immune cells that were not killed by radiation often remains intact [118, 119] and that good anti-tumor immune responses can be achieved even by irradiation of tumors that show a high immune cell infiltration. It was hypothesized, as already mentioned above, that the regional lymph nodes are of high importance to assure sufficient supply tumor-specific T cells [120]. Future clinical studies with radiotherapy in combination with immune therapies should focus on such challenges to efficiently adapt dose and volume in radiotherapy with respect to radiation-induced immune alterations.

As meaningful for multimodal tumor therapies, “multimodal” biomarkers, matrices of biomarkers, taking into account genetics, proteomics, and “immunomics” should be considered, as already addressed in the cancer immunogram by Blank et al. [101]. By next-generation sequencing, e.g., a correlation between mismatch repair deficiency and the level of tumor mutational burden (TMB) can be deduced. The latter was the reason for the FDA to approve pembrolizumab (anti-PD-1) for solid tumor independently of the tumor entity [121]. However, the response to combination of radiotherapy and immunotherapy is not only a matter of genetic mutations; it is also connected to numerous immune cell actions. In a case study of a 33 year old melanoma patient who was treated with immunotherapy, immunophenotyping of peripheral blood revealed that HLA-DR expression was increased on monocytes upon additional radiotherapy, while the number of myeloid-derived suppressor cells (MDSCs) decreased [122]. The multicolor flow cytometry (MFC) is a very charming technique for immunophenotyping, because only a few milliliters of whole blood are sufficient to analyze immune cells and their subpopulations including the activation status of the cells [15]. High expression of HLA-DR on monocytes was shown to be connected to responses to anti-PD-1 immunotherapy of patients with melanoma. Here, responding patients additional showed a higher infiltration rate of CD8^+^ T cells as confirmed in biopsies [123]. Future translational research should strongly focus on complementary analyses of immune markers in the tumor in interconnection with the immune status in the peripheral blood. The latter can easily be monitored at multiple time points without any significant additional burden for the patients. This also applies for available diagnostic- and treatment planning-related imaging data sets generated by computed tomography or magnetic resonance imaging. With radiomic approach associations between qualitative and quantitative information extracted from clinical images, clinical data and immune status can be revealed. It was demonstrated just recently that such a radiomic approach is feasible to assess tumor-infiltrating CD8 cells and response to anti-PD-1 or anti-PD-L1 immunotherapy [124]. Currently, such data sets are also analyzed for patients who do receive immunotherapy in combination with radiotherapy.

**Fig. 2:**
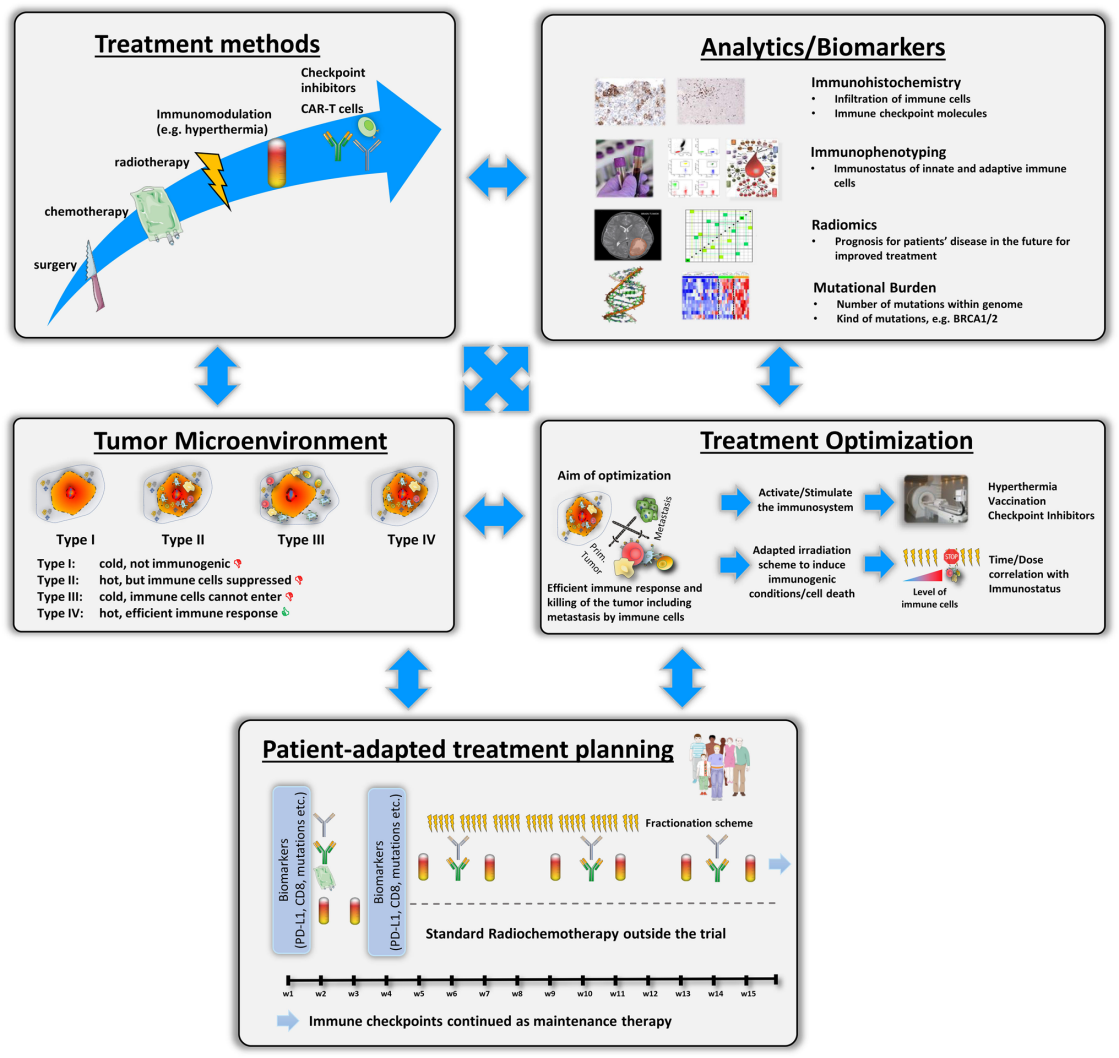
Considerations for well-elaborated patient-adapted tumor treatment protocols. The outcome of conventional treatment methods, i.e., surgery and radio(chemo)therapy, can be improved by additional immunomodulation with, e.g., hyperthermia, CAR-T cells and immune checkpoint inhibitors. Furthermore, joint and innovative analytical techniques like immunohistochemistry, immunophenotyping, radiomics, and the detection of mutational burden might help to find out patient-specific properties and should be the basis for treatment optimization. One key aim in treatment optimization is to turn cold tumors (type I) into hot tumors (type IV) and to eliminate barriers of immune-suppressed or immune-excluded (type II and III) tumors. Finally, all parameters should be used for patient-adapted treatment planning and testing within clinical trials

As shown by Multhoff et al. in pre-clinical models of glioblastoma and lung cancer, inhibition of PD-1 in combination with ex vivo HSP70-activated NK cells significantly prolonged overall survival by a factor of 2.3 in comparison to control animals or monotherapy [125]. A correlation between tumor size and HSP70 was also found for non-small cell lung cancer [126]. Therefore, additive methods such as hyperthermia and vaccination could further boost anti-tumor immune responses related to combined treatments with radiotherapy and/or chemotherapy and immune checkpoint inhibition [127]. Figure 2 summarizes the key and manifold challenges for optimized patient-adapted treatment planning with the aim to achieve both local and systemic tumor control and eradication. Besides biomarker-based improvement of anti-tumor responses, reduction of side effects should always be in the focus. This is already followed up in clinical trials as described above for the CheckRad-CD8 study and should be expanded to additional treatments as hypothetically suggested in Fig. 2.

## Abbreviations

ATP: Adenosine triphosphate
ccRCC: Clear cell renal cell carcinoma
CT: Chemotherapy
CTLA-4: Cytotoxic T-lymphocyte associated protein 4
DAMPs: Damage-associated molecular patterns
DSB: Double-strand breaks
FDA: U.S. Food and Drug Association
HHP: High hydrostatic pressure
HMGB1: High-mobility group box I protein
HSP70: Heat shock protein 70
ICC: Immunocytochemistry
ICD: Immunogenic cell death
ICI: Immune checkpoint inhibitors
ICM: Immune checkpoint molecules
IF: Immunofluorescence
IPT: Immunophenotyping
MCP: Microenvironment cell populations
MFC: Multicolor flow cytometry
NCT: National Center for Tumor Diseases
NSCLC: Non-small cell lung cancer
PCD: Programmed cell death
RONS: Reactive oxygen or nitrogen species
RT: Radiotherapy
TAM: Tumor-associated macrophages
TLS: Tumor-adjacent lymphoid islets
TMB: Tumor mutational burden
